# Fungal species in endodontic infections: A systematic review and meta-analysis

**DOI:** 10.1371/journal.pone.0255003

**Published:** 2021-07-22

**Authors:** Alice Alberti, Stefano Corbella, Silvio Taschieri, Luca Francetti, Kausar Sadia Fakhruddin, Lakshman Perera Samaranayake

**Affiliations:** 1 Department of Biomedical, Surgical and Dental Sciences, Università degli Studi di Milano, Milan, Italy; 2 IRCCS Istituto Ortopedico Galeazzi, Milan, Italy; 3 Institute of Dentistry, I. M. Sechenov First Moscow State Medical University, Moscow, Russia; 4 College of Dental Medicine, University of Sharjah, Sharjah, UAE; 5 The University of Hong Kong, Hong Kong Special Administrative Region, Hong Kong, China; Manipal College of Medical Sciences, NEPAL

## Abstract

Fungal infections are common on oral mucosae, but their role in other oral sites is ill defined. Over the last few decades, numerous studies have reported the presence of fungi, particularly *Candida* species in endodontic infections, albeit in relatively small numbers in comparison to its predominant anaerobic bacteriome. Here, we review the fungal biome of primary and secondary endodontic infections, with particular reference to the prevalence and behavior of *Candida* species. Meta-analysis of the available data from a total of 39 studies fitting the inclusion criteria, indicate the overall weighted mean prevalence (WMP) of fungal species in endodontic infections to be 9.11% (from a cumulative total of 2003 samples), with 9.0% in primary (n = 1341), and 9.3% in secondary infections (n = 662). Nevertheless, WMP for fungi in primary and secondary infections which were 6.3% and 7.5% for culture-based studies, increased to 12.5% and 16.0% in molecular studies, respectively. The most prevalent fungal species was *Candida* spp. The high heterogeneity in the reported fungal prevalence suggests the need for standardized sampling, and speciation methods. The advent of the new molecular biological analytical platforms, such as the next generation sequencing (NGS), and matrix-assisted laser desorption ionization time-of-flight mass spectrometry (MALDI-TOF), that enables identification and quantitation of a broad spectrum of hitherto unknown organisms in endodontic infections should radically alter our understanding of the endodontic mycobiome in the future. *Candida* spp. appear to be co-pathogens with bacteria in approximately one in ten patients with endodontic infections. Hence, clinicians should comprehend the importance and the role of fungi in endodontic infections and be cognizant of the need to eradicate both bacteria and fungi for successful therapy.

## Introduction

Fungi are common opportunistic pathogens, and they comprise a substantial proportion of the human microbiome [1]. The fungal human microbiome, termed the mycobiome has numerous constituents, but the predominant of which belongs to the genus *Candida* [2]. Of over 200 species of human pathogenic and saprophytic, *Candida* species the following, which are of major medical importance occupy the top positions in the hierarchy of the *Candida* biome: *C*. *albicans*, *C*. *glabrata*, *C*. *kefyr*, *C*. *krusei*, *C*. *parapsilosis* and *C*. *tropicalis*. Of these, *C*. *albicans* is, by far, the commonest intra oral species, in 30–45% healthy adults, and the main agent of oral candidal infections [3]. Two reviews of the microbiology of endodontic infections reported *C*. *albicans* as the most common isolate from the endodontic mycobiome [4,5].

The transition of *Candida* from a harmless oral commensal to an opportunist pathogen depends on a number of predisposing conditions, including immune functionality, endocrine disorders, ill-fitting dentures, poor oral hygiene, use of broad-spectrum antibiotics, corticosteroids, immunosuppressive agents, and drugs that may induce neutropenia and xerostomia [3]. *Candida* species, in general possess an armamentarium of virulence attributes which, acting in concert may cause candidiasis in vulnerable individuals when an opportunity arises [1,6,7]. These include i) adhesion and biofilm formation on biotic and abiotic host surfaces, mediated by surface molecules [6]; ii) production of hydrolytic enzymes, proteinases, phospholipases, and hemolysins which degrade extracellular matrix proteins of the host tissues [6,7]; iii) thigmotropism; iv) phenotypic switching and consequent environmental adaptability; v) evasion of host immune system, through degradation of IgG1, IgA1, and IgA2 [6,7] and suppressing polymorphonuclear neutrophil functions; and vi) immunomodulation through stimulation of proinflammatory cytokine synthesis and activation of the complement cascade [6].

*Candida* spp. have a predilection to reside in a number of specific oral niches. The dorsum of the tongue is considered its primary oral habitat [1] although the fitting surface of acrylic denture surface is the major reservoir of the yeasts in denture wearers. Nevertheless, it can habituate other oral mucosal sites, as well as supra- and sub-gingival plaque biofilms [8,9], periodontal pockets [10], carious lesions and infected root canals (*syn*. endodontium) [6].

The role of *Candida* spp. in carious and endodontic infections is ill defined. However, there is a growing body of data to implicate that the *Candida* biome play a significant role in the pathogenesis of dental caries and the resultant endodontic sequelae [11–13], as discussed below.

In a seminal recent review, Pereira et al. [14] have elegantly argued the plausible basis of the cariogenic potency of *Candida*. In clinical terms, the isolation of *Candida* spp. from enamel, dentine and root caries [15], the correlation between high salivary *Candida* carriage and the severity of caries [16,17], and its high prevalence in early childhood caries [18] demonstrate that *Candida* spp. may have a significant impact on the carious process.

Further examination of the pathogenic attributes of *Candida* spp. that define it as a candidate cariogen is salutary. For instance, *in vitro* studies reveal that *C*. *albicans* is able to firmly adhere to normal or EDTA/NaOCl-treated enamel, dentine, cementum surfaces [19,20], and it has a very high affinity for hydroxyapatite [19,21,22]. The presence of the smear layer increases the adhesion of *C*. *albicans* to human dentin [23,24], probably due to the availability of exposed dentinal collagen and calcium ions. Indeed, *Candida* is able to bind to collagen types I and IV [6,25–27], and it has a specific affinity for dentinal collagen. Furthermore, calcium ions modify *Candida* morphogenesis and its capacity to adhere to extracellular matrix proteins [23,24,28]. Due possibly to its thigmotrophic properties, *C*. *albicans* has the unique ability to penetrate deep into exposed dentinal tubules in carious lesions. For these reasons, some have called the yeast a “dentinophilic” microorganism [29]. In terms of community living within cariogenic niches, many *Candida* spp. have the potential to co-aggregate with cariogenic bacteria [6,14]. Interestingly, Falsetta et al. [30] reported that *C*. *albicans* induces virulence gene expression in *S*. *mutans*, that in turn may facilitate bacterial-yeast aggregation.

Many *Candida* spp. can rapidly metabolize dietary carbohydrates such as glucose, sucrose, fructose, and even polyols (e.g. xylitol) [14], leading to the formation of acidic end-products such as short-chain carboxylic acids, lactate and acetates that demineralize enamel. The acidification is also due to the activation of H+-ATPase-dependent sugar uptake system of *C*. *albicans* plasma membrane, and to the generation of carbon dioxide from glucose metabolism [31]. Such acid production in a localized, focal biofilm niche causes a rapid drop of the pH to 5.5 or below leading to enamel and dentine demineralization [14,19,32]. The reduction of pH also leads to the activation of acid proteases, collagenases, and phospholipases of *Candida* spp., that assist degradation of the dentinal collagen [25,26,33,34]. In conclusion, *Candida* could have a deleterious cariogenic effect on dental hard tissues through independent, dichotomous mechanisms: first, by dissolving the inorganic matrix of either enamel or dentine with its metabolic acids, and second, by disassembling the organic matrix by its abundant collagenolytic enzymes.

There are also a number of studies that points to a critical role of *Candida* in the pathogenesis of endodontic infections. The yeast, being a microaerophilic eukaryote possesses the metabolic armory necessary to survive within the harsh and barren ecosystem of the root canal. Several workers have shown that *C*. *albicans* could use dentin itself as a source of nutrition *in vitro*, in the absence of other extraneous food supplements, and colonize the canal walls as well as the dentinal tubules [29,35,36]. In one experiment, the penetration of *C*. *albicans* into dentinal tubules *in vivo* was shown to be facilitated by the presence of a smear layer, produced by instrumentation [37]. The sheltered life of the yeast within the tubules, out of reach of instruments, and disinfectant irrigants, is also likely to perpetuate chronic endodontic infections [6,38].

With regard to the localization of fungi within the root canal system, Nair et al. [39] elegantly demonstrated through ultrastructural imaging, the presence of yeast-like organisms in root canals, and at the apical foramen. Persoon et al. [40] also observed dense masses of yeast cells in root canals, while dentinal tubules were filled with hyphae. Siqueira et al. [41] investigated the patterns of microbial colonization in primary root canal infections through scanning electron microscopy, and observed single or budding yeast cells in root canal systems.

Nevertheless, since then, there has been an explosive advancement in microbial identification techniques, and new data are available on the fungi in this oral eco-niche. Hence, the aim of this systematic review and meta-analysis was to review the recent literature on the prevalence of fungal species in primary and secondary endodontic infections.

## Materials and methods

### Data sources

Two investigators (AA and SC) performed an electronic search of MEDLINE/PubMed, SCOPUS, ISI Web of Science, and Cochrane Central without any language restrictions published since 2000. Grey literature was searched for pertinent papers by using OpenGrey and Greylist. A manual search was performed of all the issue published since 2000 of *Journal of Endodontics*, *International Endodontic Journal and Oral Surgery*, *Oral Surgery*, *Oral Medicine*, *Oral Pathology*, *Oral Radiology*. Moreover, reference lists of all included papers were screened for articles that need to be evaluated for inclusion in the study. The final electronic search was performed on 10^th^ March 2021. Our investigation was focused on relatively recent literature, in view of the fact that older, conventional microbiological analytical techniques, mainly depending on phenotypic analyses, have undergone a radical revolution with the advent of molecular biological screening techniques, such as next generation sequencing (NGS) platforms.

The specific review question formulated was as follows: What is the prevalence of fungal species in primary and secondary endodontic infection in human permanent teeth?.

### Search terms

A specific search string was formulated for each of the databases, which included each of the following group of search terms, combined by means of the Boolean operator AND:

- (Dental pulp disease?) OR (apical periodont*) OR (periapical periodont*) OR (apical disease?) OR (periapical disease?) OR (periapical lesion?) OR (apical lesion?) OR pulpitis OR (dental pulp necrosis) OR (tooth necrosis) OR (endodontic lesion?) OR (endodontic pathosis).
- Fung* OR mycet* OR mycos* OR candid* OR yeast? OR microb*
- Exp dental pulp diseases OR exp periapical diseases OR exp periapical periodontitis OR Exp fungi

### Study selection

#### Inclusion criteria

Human endodontic studies; studies on the detection of fungal species within infected root canal of either carious teeth or after failed endodontic treatment; studies clearly relating method of preparation of the sampling site and sample collection; clear description of the methodology employed for fungal detection; use of standardized methods for sample collection, transportation, and analysis (e.g., isolation with a rubber dam, use of sterile paper point; studies on permanent teeth with complete root formation.

#### Exclusion criteria

Exclusion criteria were *ex vivo* and *in vitro* studies and *in vivo* studies in teeth with endo-periodontal lesions.

### Electronic data-search and assessment

To answer the review question, a four-step approach of evidence-based analysis was employed. According to the pre-set inclusion and exclusion criteria, the title and abstract were independently screened by two authors (AA and SC) at stage-one; full texts of the included records were assessed for eligibility by the same authors (AA and SC). Disagreements in article selection processes were solved by consulting a third reviewer (LS). At stage-two of the data extraction, the author (AA) meticulously screened the included full texts and extract information on sample size, primary or secondary infection after endodontic treatment. Each study’s characteristics were recorded employing the Cochrane pattern determining the study design, setting, methods sample collection, culture medium, yeast species recovered. Additionally, the microbiological evaluation and outcomes about the prevalence of fungal species were recorded in a spreadsheet. The authors were contacted via emails if insufficient information were available from the included studies.

#### Quality of the evaluated studies

At the third stage of the review, a critical appraisal of the included studies was performed adopting a modification of the checklist proposed by Hoy and colleagues [42] using the following criteria: i) was the target population close representation of the national population in relation to relevant variables? ii) was the sampling frame a true or close representation of the target population? iii) were data collected directly from the subjects? iv) was the study instrument that measured the parameter of interest shown to have reliability and validity? v) was the same mode of data collection used for all subjects? Based on the above criteria a score was assigned for each study. A score of 0 is low risk bias, while (1–2) is considered as moderate risk and a score between (3–5) reflects high risk of bias, respectively.

### Summary measures, synthesis of results, and other analysis

The fourth and final stage involved the analysis of the results, performed by an author (SC) by using R (R Core Team (2013). R: A language and environment for statistical computing. R Foundation for Statistical Computing, Vienna, Austria. URL http://www.R-project.org/).

Data on the prevalence of fungal species were extrapolated from the studies and pooled together by calculating the weighted mean prevalence (based on the number of samples in each study). Separate analyses were performed for i) primary and the secondary endodontic treatment; ii) methods used for evaluating the presence of fungal species (molecular, cultural, or both), iii) the collection method (e.g., paper points and endodontic hand files), and iv) studies with periapical lesions in systemically healthy subjects. The meta-analysis was performed using the DerSimonian-Laird random-effects method (assuming heterogeneity among studies), calculating pooled proportion.

Cochran’s test served to measure the results’ consistency, with a significant level set at *P* <0.1. The heterogeneity of the outcomes was also evaluated as follows: <40% the heterogeneity was considered negligible, 41% to 60% moderately heterogeneous, if 61% to 90% substantially heterogeneous [43].

## Results

**Fig 1:**
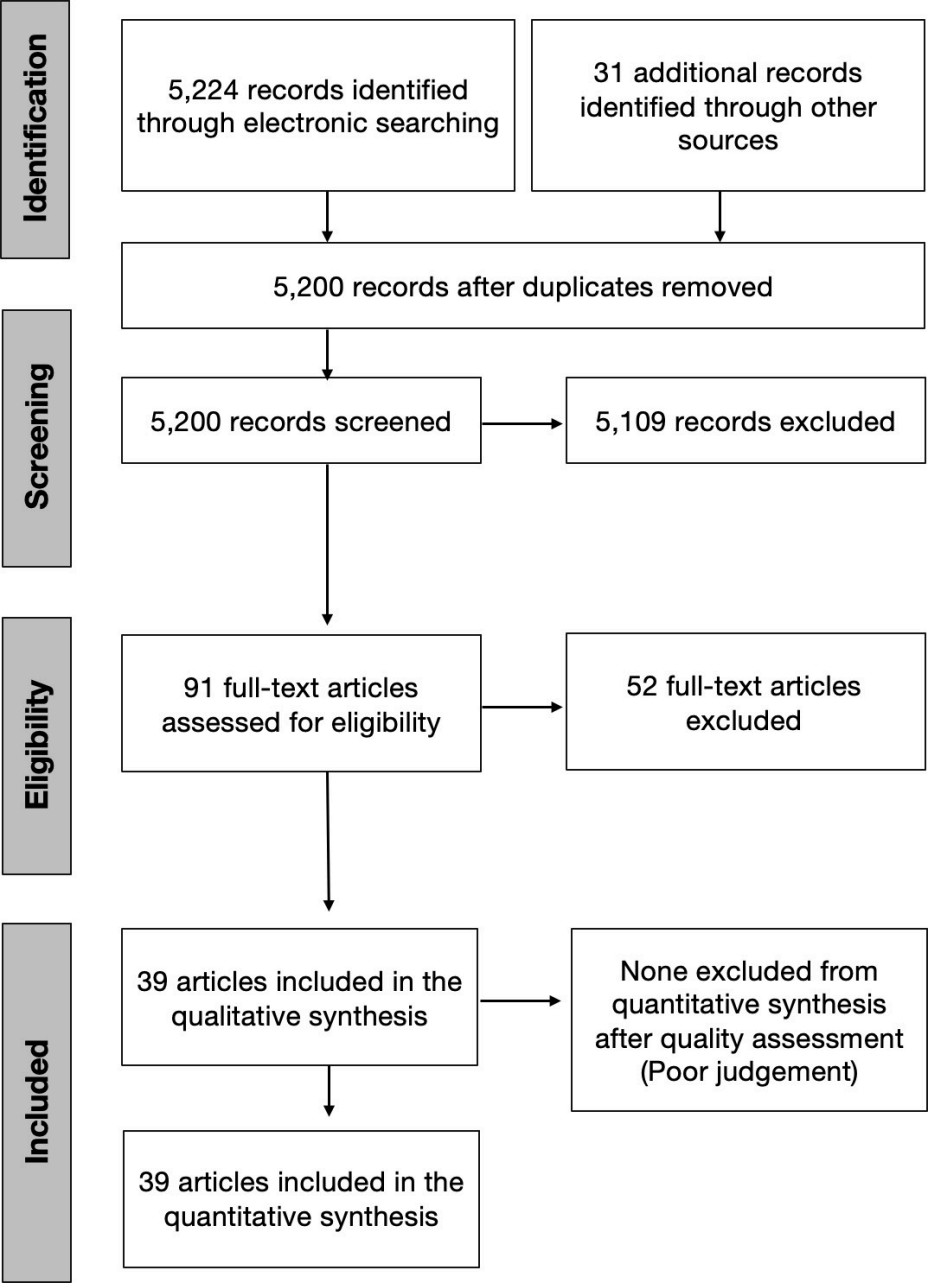
The review search and selection flowchart.

**Fig 2:**
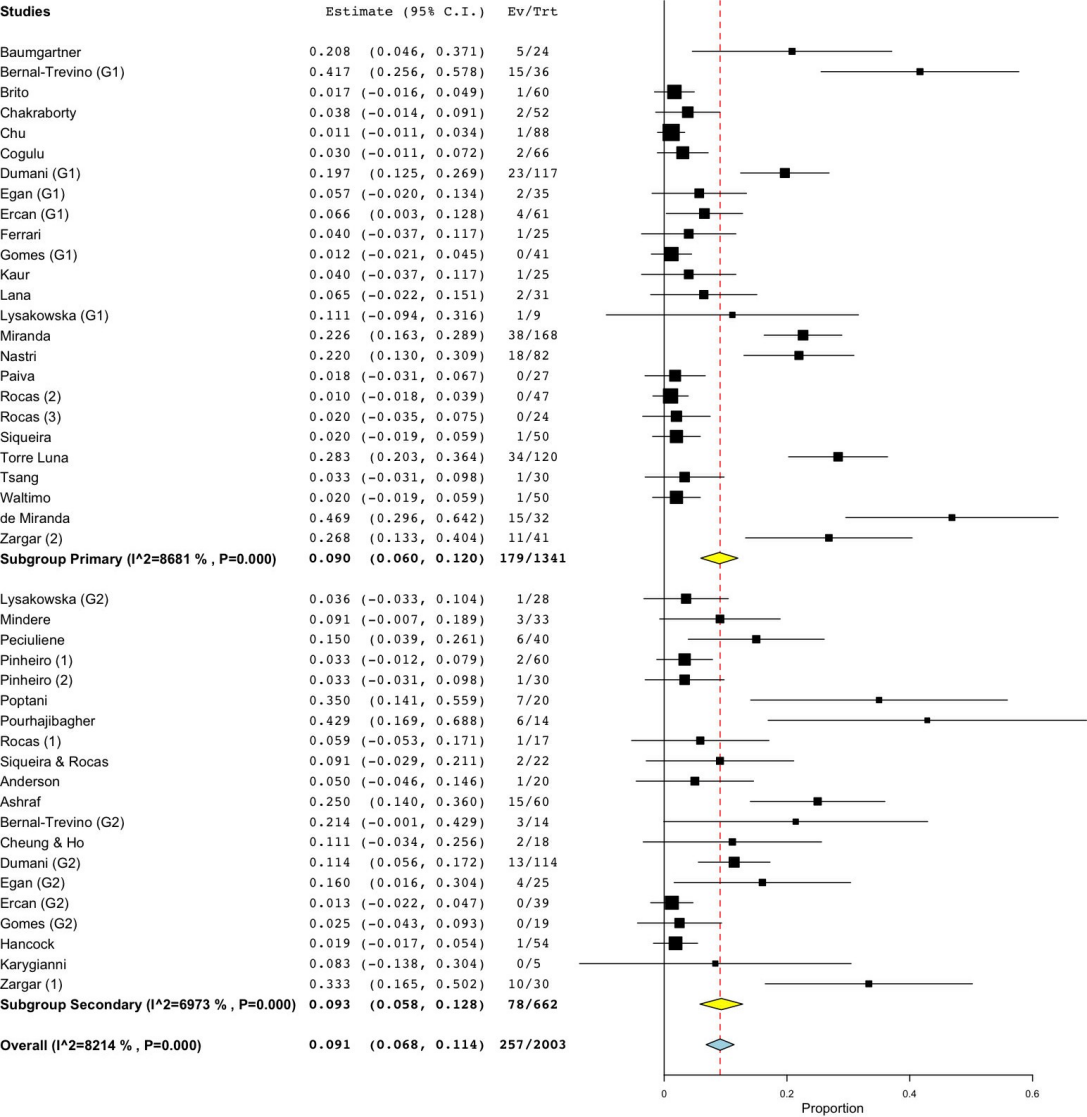
Forest plot representing fungal species prevalence in included studies, distinguishing primary or persistent lesions.

The initial search identified 5,255 articles. Among them, 55 were duplicates and hence excluded. Of the remaining 5,200 papers, 5,110 were excluded after perusal of the titles and abstracts as they did not fit the inclusion criteria. Further screening for full texts resulted in the exclusion of 52 articles, as the outcomes were incongruent with the study objectives. Finally, a total of 39 studies using culture and molecular fungal assessment methods were included in the current review [44–82]. Twenty-three included studies used the culture method for fungal evaluation. Among them, eight studies examined samples from primary endodontic infection, while ten, assessed the presence of fungal species in teeth with secondary endodontic infection. Another five studies evaluated the fungal presence in either primary or secondary endodontic infections. Of nineteen molecular studies reviewed, 12 studies examined the fungal presence in primary endodontic infection, while six appertained to secondary infection, and a single study in either a primary or a secondary infection. Fig 1 represents the articles selection process. Tables 1–6 present the characteristics of the included studies which investigated the yeast colonization of root canals in primary and secondary pulp infections through cultural methods and molecular analytical techniques, respectively. The prevalence of fungi in primary endodontic infections was investigated in 25 studies, with a total of 1341 analyzed samples and a weighted mean prevalence (WMP) of 9.0% (CI 95%: 6.0–12.0) (Fig 2). We found a significant heterogeneity among papers with regard to the reported values, as the prevalence of fungal species varied between 0 and 46.8% [78].

**Table 1:** Characteristics of the included studies using only culture methods for yeast identification, from primary endodontic infections.

Primary Endodontic Infection (culture method)							
Study Country	No. of subjects and characteristics	No. of samples and characteristics	Procedures before collection	Sample collection	Culture medium Yeast identification method	Yeast spp. recovered (number of cases; prevalence)	Risk of bias
**Lana et al. 2001** [46] **Brazil**	NS	31 Single-rooted teeth	Isolation; disinfection (30% H2O2, 5% iodine, 5% Na2S2O3), sterility control samples; endodontic access; irrigation with saline	Paper points for 1 min; placed in PRAS Ringer solution	Sabouraud agar + chloramphenicol (100 mg/ml) plates Germ tube, morphology, carbohydrate assimilation, carbohydrate fermentation	*C*. *tropicalis* (2; 6.45%)	0
**Chakraborty et al. 2005** [56] **India**	NS	52 Non-vital teeth; no direct communication between pulp and oral cavity; no deep periodontal pockets	Cleansing with pumice paste, rubber dam isolation, disinfection (70% isopropyl alcohol for 2 min, povidone-iodine 7.5% for 6 min); endodontic access. Saline solution irrigation.	Paper point; placed in thioglycolate broth. Processed within 2 h	Nutrient Agar, Blood Agar, Chocolate agar, MacConkey’s Agar Colony morphology, Gram staining, motility test, biochemical test, sugar fermentation reactions	*C*. *albicans* (2; 3.85%)	0
**Chu et al. 2005** [57] **Hong Kong**	87 no uncontrolled systemic diseases; no antibiotic cover; age range 16–71 yrs	88 Periapical radiolucency, non-vital, single and multirooted (only the largest canal with periapical lesion was sampled), with and without communication with oral cavity	Cleansing with pumice, rubber dam isolation, disinfection (4% CHX gluconate for 3 min, 10% iodine for 1 min, 5% Na2S2O3), caries/restoration removal with bur + sterile saline irrigation, disinfection procedures repeated	Paper points for 15–20 s, placed in RTF	SDA Colony morphology, germ tube test, biochemical test, chromogenic *Candida* differential agar	*C*. *albicans* (1; 1.14%)	0
**Ferrari et al. 2005** [58] **Brazil**	25 No systemic diseases; age range 23–49 yrs; different races; no antibiotic in previous 3 months	25 Single-root teeth with asymptomatic periapical radiolucency; no communication with oral cavity, no fistula	Mouthwash (0.12% CHX for 60 s), rubber dam isolation, disinfection (30% H2O2, 10% iodine tincture, 5% Na2S2O3, 60 s each), sterility control samples (cotton pellet); endodontic access (with water spray)	3 paper points for 30 s; placed in VMGA III; average time before processing: 6 h	SDA + 0.1% chloramphenicol Colony morphology, Gram staining, biochemical test	*Candida* spp. (1; 4%)	0
**Waltimo et al. 2005** [59] **USA**	NS	50 chronic apical periodontitis	Rubber dam isolation, disinfection (0.12% CHX gluconate for 1 min), sterility control samples, endodontic access	Paper points; processed within 2 h	Sheep blood cell agar Biochemical test	*C*. *albicans* (1; 2%)	0
**Nastri et al. 2011** [66] **Argentina**	82 Immunocompetent patients, age range 18–70 yrs; no systemic diseases, no severe periodontitis; no antibiotics/NSAIDs/corticoids/anti-fungal medications	82 upper incisors; periapical radiolucency, crown-root integrity, no restorations, asymptomatic	Rubber dam isolation, 10% povidone iodine, endodontic access	Paper point; placed in PBS	Solid chromogenic differential medium for *Candida* spp. Color analysis, development of pseudomycelium with chlamydoconidia in 1%-Tween 80 milk agar, carbohydrate assimilation	Total fungal species (18; 21.95%) *C*. *albicans* (5; 6.10%), *C*. *dubliniensis* (9; 10.98%); *C*. *guilliermondii* (2; 2.44%); *C*. *krusei* (1; 1.22%); *C*. *tropicalis* (1; 1.22%)	0
**Paiva et al. 2012** [72] **Brazil**	30 no antibiotics in the last 3 months, no periodontitis	27 (3/30 excluded for positive sterility control samples) Single-rooted teeth, asymptomatic periapical lesion, no periodontal pocket, no gross carious lesion/fracture	Pumice cleansing, caries/restoration removal, rubber dam isolation, disinfection (6% H2O2, 2% iodine, 6% H2O2, 2.5% NaOCl) endodontic access (sterile saline irrigation), disinfection (as above + 5% Na2S2O3); sterility control samples (paper points). No chemical irrigants. Introduction of sterile saline into the canals	5 paper points for 1 min; 2 samples placed in thioglycolate broth, 1 in phosphate-buffered saline. Inoculated in CHROMagar), (other samples for PCR)	CHROMagar Selective media	Yeasts: 0	0
**Kaur et al. 2014** [74] **India**	25 Age range 20–40 yrs	25	NS	Paper point for 1 min; placed in saline. Processed within 24 h	SDA Gram staining, Germ tube test, chromogenic *Candida* differential agar	*C*. *albicans (1; 4%)*	2

BHI = brain heart infusion; CBA = Columbia blood agar; CHX = chlorhexidine; ETSA = enriched tryptic soy agar; F = females; FAA = fastidious anaerobe agar; h = hour(s); H2O2 = hydrogen peroxide; HCB = yeast–cysteine blood agar; LDTM = liquid dental transport media; M = males; Min = minutes; Na2S2O3 = sodium thiosulfate; NaOCl = sodium hypochlorite; NS = not specified; NSAIDs = Nonsteroidal anti-inflammatory drugs; PBS = Phosphate-buffered saline; PRAS = pre-reduced anaerobically sterilized; R2A agar = Reasoner’s 2 agar; RCT = root canal treatment; RTF = reduced transport fluid; s = seconds; SDA = Sabourad dextrose agar; SDS-PAGE = sodiumdodecylsulphate polyacrylamide gel electrophoresis; spp. = species; TSBV = Tryptic Soy-serum-Bacitracin-Vancomycin; VMGA = Viability Medium Göteborg Agar; yrs = years. **RISK OF BIAS**: (0 low risk; 1–2 moderate risk; 3–5 high risk).

**Table 2:** Characteristics of the included studies using only culture methods for yeast identification, from secondary endodontic infections.

Secondary Endodontic Infection (culture method)							
Study Country	No. of subjects and characteristics	No. of samples and characteristics	Procedures before collection	Sample collection	Culture medium Yeast identification method	Yeast spp. recovered (number of cases; prevalence)	Risk of bias
**Hancock et al. 2001** [45] **United States**	54 (age range 15–82 yrs, M: F = 32:22)	54 Periapical radiolucency; RCT completed at least 3 yrs earlier; no direct exposure to the oral cavity	Rubber dam isolation, disinfection (iodine); endodontic access. Post-removal with ultrasonic vibration or sterile bur, disinfection (30% H2O2, iodine, 5% Na_2_S_2_O_3_); sterility control samples (cotton pellet moistened in 5% Na_2_S_2_O_3_), mechanical root-filling removal (no solvent). LDTM into the canal	(a) Paper points. (b) K-type files (+ saline solution irrigation); placed in LDTM	NS Colony morphology, Gram staining, micromorphology, physical and biochemical tests, selective media	(a) *C*. *albicans* (1, 1.85%; not associated with tested bacterial spp.) (b) 0	0
**Peciuliene et al. 2001** [47] **Lithuania**	40 No systemic diseases; no antibiotics in the last 2 months	40 Periapical radiolucency, RCT completed 5–10 yrs earlier	Rubber dam isolation; mechanical removal of root filling	Paper points; placed in VMGA III gel; cultivated 24–48 h after sampling	Sabouraud plates; TSBV agar Gram staining, production of glycosidase enzymes, comparison of silver-stained whole cell protein profiles of the isolates in SDS-PAGE with reference strains	*C*. *albicans* (6; 15%; always associated with bacteria, particularly with *E*. *faecalis* in 3 cases)	1
**Cheung & Ho 2001** [48] **Hong Kong**	18 Southern Chinese, age range 18–73 yrs	18 Asymptomatic periapical radiolucency; RCT completed at least 4 yrs earlier; no communication with oral cavity, no periodontal pockets	Rubber dam isolation, disinfection (30% H2O2), restoration removal, disinfection (30% H2O2, 10% ethanolic iodine for 1 min, 5% Na_2_S_2_O_3_), check of the sterility swabbing a cotton pellet; endodontic access, no chemical irrigant used; RTF inside canal	3–4 paper points; placed in RTF, 1 placed in liquid thioglycollate medium; 1 unused paper point as negative control; processed within 10 min	ETSA; MacConkey agar; SDA. Phase-contrast microscopy, morphotyping, Gram staining, biochemical tests, micromorphology, colony morphology, selective media	*C*. *albicans* (2; 11.11%)	0
**Pinheiro et al. 2003** [51] **Brazil**	NS No systemic diseases, no antibiotics in the last 3 months	60 Periapical radiolucency, RCT completed at least 2 yrs earlier. Only 1 canal per tooth sampled (the largest with periapical lesion)	Restoration removal, rubber dam isolation, disinfection (5.25% NaOCl, 5% Na_2_S_2_O_3_), endodontic access, chemical irrigants not used, saline to moisten the canal	Paper point for 60 s; the canal orifice flushed with nitrogen gas during sampling; placed in VMGA III; average time processing 4 h	5% defibrinated sheep blood-FAA, 5% defibrinated sheep blood- Columbia agar plates Gram staining, catalase production, gaseous requirements	*Candida* spp. (2; 3.33%)	0
**Pinheiro et al. 2003** [52] **Brazil**	NS No systemic diseases, no antibiotics in the last 3 months	30 Periapical radiolucency, RCT completed at least 4 yrs earlier. Only 1 canal per tooth sampled (the largest with periapical lesion)	Restoration removal, rubber dam isolation, disinfection (30% H2O2, 5.25% NaOCl, 5% Na_2_S_2_O_3_), endodontic access, chemical irrigants not used, saline to moisten the canal	Paper point for 60s; the canal orifice flushed with nitrogen gas during sampling; placed in VMGA III; average time processing 4 h	Sabouraud agar + 100 μg/ml of chloramphenicol Gram staining, catalase production, gaseous requirements, biochemical test	*C*. *albicans* (1; 3.33%)	0
**Ashraf et al. 2007** [61] **Iran**	NS no systemic diseases; no long-term use of antibiotics/corticosteroids	60 Molars; no coronal leakage; 30 with periapical lesions	Polishing cup, rubber dam isolation, disinfection (10% iodine), saline; removal of restoration, disinfection (10% iodine), surgical gloves, mechanical removal of root filling	#20 Headstrom files, placed in TS broth; processed within 3 h	Blood agar, MacConkey agar, then SDA Colony morphology, diagnostic test	With periapical lesion: *C*. *albicans* (11; 36.7%). Without periapical lesion: *C*. *albicans* (4; 13.3%). Total: *C*. *albicans* (15; 25%)	0
**Mindere et al. 2010** [65] **Latvia**	NS no antibiotics in the last 3 months, no systemic diseases	33 Periapical radiolucency; improper RCT, performed >4 yrs before; no acute periapical pathology, no sinus tract, no temporary filling/missing restorations	Access cavity preparation, rubber dam isolation, disinfection (5.25% NaOCl, 5% Na_2_S_2_O_3_), endodontic access, mechanical removal of root filling	Paper point for 1 min (from coronal and apical part of the root)	R2A agar, sheep blood agar Micromorphology	Fungal species (4 isolates, 3 cases; 9.09%), *C*. *albicans* (2; 6.06%), *Saccharomyces* spp. (1; 3.03%), *Cryptococcus* spp. (1; 3.03%)	0
**Anderson et al. 2012** [69] **Germany**	21 no systemic diseases, no antibiotics in the last 30 days	20 (1/21 excluded for contamination of quality control sample) RCT completed at least 2 years earlier, no direct exposure to oral cavity, asymptomatic	Rubber dam isolation, disinfection (30% H2O2, 2,5% NaOCl), endodontic access, disinfection ((30% H2O2, 2,5% NaOCl, 5% Na_2_S_2_O_3_), sterility control samples (foam pellets), mechanical removal of root filling (no solvent). Introduction of sterile saline into the canals	3 paper points for 1 min; placed in RTF, frozen at -20°C	HCB, CBA, bile esculin plates Gram staining, cell morphology, biochemical tests	Fungal species 0	0
**Karygianni et al. 2015** [75] **Germany**	5 No periodontitis, no severe systemic diseases, no antibiotics in the last 30 days	5 First lower molars, asymptomatic, RCT completed at least 2 yrs earlier, no direct exposure to oral cavity	Disinfection (30% H2O2, 2.5% NaOCl), rubber dam isolation, endodontic access, disinfection (30% H2O2, 2.5% NaOCl, 5% Na_2_S_2_O_3_), sterility control sample, mechanical root-filling removal (no solvents), introduction of sterile saline into the canal	(a) Obturation material samples; placed in RTF (b) 3 paper points for 1 min; placed in RTF and frozen at -80°C	CBA, HCB, and bile esculin plates Gram staining, cell morphology, biochemical tests	Fungal species 0	1
**Pourhajibagher et al. 2017** [77] **Iran**	14 No severe systemic disease, no antibiotics in the previous 1 month	14 Single-rooted teeth, RCT performed > 2 yrs earlier	Pumice cleansing, removal of caries/restorations/post, rubber dam isolation, disinfection (30% H2O2 for 30s, 2.5% NaOCl for 30 s, 5% Na_2_S_2_O_3_), root-filling removal (no chemical solvent)	3 paper points for 60 s, placed in VMGA III, processed within 4 h	SDA + 100 μg/mL chloramphenicol Colony morphology, Gram staining, antibiotic susceptibility, biochemical test	*C*. *albicans* (6; 42.86%)	0

BHI = brain heart infusion; °C = degree Celsius; CBA = Columbia blood agar; CHX = chlorhexidine; ETSA = enriched tryptic soy agar; F = females; FAA = fastidious anaerobe agar; H2O2 = hydrogen peroxide; HCB = yeast–cysteine blood agar; LDTM = liquid dental transport media; M = males; Min = minutes; Na2S2O3 = sodium thiosulfate; NaOCl = sodium hypochlorite; NS = not specified; NSAIDs = Nonsteroidal anti-inflammatory drugs; PBS = Phosphate-buffered saline; PRAS = pre-reduced anaerobically sterilized; R2A agar = Reasoner’s 2 agar; RCT = root canal treatment; RTF = reduced transport fluid; S = seconds; SDA = Sabourad dextrose agar; SDS-PAGE = sodiumdodecylsulphate polyacrylamide gel electrophoresis; TSBV = Tryptic Soy-serum-Bacitracin-Vancomycin; VMGA = Viability Medium Göteborg Agar. **RISK OF BIAS**: (0 low risk; 1–2 moderate risk; 3–5 high risk).

**Table 3:** Characteristics of the included studies assessing prevalence of yeasts in teeth with primary and secondary endodontic infection using culture methods.

Study Country	No. of subjects and characteristics	No. of samples and characteristics	Procedures before collection	Sample collection	Culture medium Yeast identification method	Yeast spp. recovered (number of cases; prevalence)	Risk of bias
**Egan et al. 2002** [49] **United Kingdom**	55 No conditions promoting *Candida* carrier state (prolonged antibiotics/steroid therapy, anemia, diabetes)	60 (35 primary; 25 secondary)	Rubber dam isolation, disinfection (30% H2O2, 10% iodine for 1 min, 5% Na_2_S_2_O_3_), endodontic access, disinfection repeated; mechanical removal of root filling + solvent (chloroform) in 3 cases. Introduction of sterile phosphate buffered saline into the canal	3 paper points for at least 1 min, placed in RTF; processed within 3 h	SDA Growth characteristics, colony morphology, Germ tube formation test, hyphal morphology, biochemical tests	*R*. *mucilaginosa*, *C*. *albicans*, *C*. *sake* (6 root canals, 10%; 5 patients, 9.1%. 2/35 primary, 5.71%. 4/25 secondary, 16%. All 5 patients had received a course of antibiotics within the previous 12 months and all positive root canals had communication with oral cavity	0
**Gomes et al. 2004** [54] **Brazil**	60 no general diseases; no antibiotics on the previous 3 months	60 (one root canal per patient, for multi-rooted teeth the canal with exudation, or largest, or associated with periapical radiolucency, 41 primary infection and 19 secondary infection (necrotic pulp tissues/RCT completed at least 4-years before)	Rubber dam isolation, disinfection (30% H2O2, 2.5% NaOCl, 5% Na_2_S_2_O_3_); endodontic access (manual irrigation with sterile saline solution). Chemically active irrigants not used. Mechanical removal of root-filling (without solvents)	Paper point for 1 min; processed within 4 h	SDA + 100 μg/ml chloramphenicol Colony morphology, Gram staining, catalase production, gaseous requirements, biochemical test	Fungal species 0	1
**Ercan et al. 2006** [60] **Turkey**	90 no antibiotics in the last 3 months; no systemic diseases	100 Single root canals. 61 primary infection 39 secondary infection	Rubber dam isolation, caries/restoration removal (manual irrigation with saline solution), disinfection (30% H2O2, 2.5% NaOCl for 30 s, 5% Na_2_S_2_O_3_), endodontic access, mechanical removal of root filling (no solvent)	Paper points for 60 s; The canal orifice was flushed with nitrogen gas during the sampling process; placed in RTF; processed within 4 h	Sabouraud agar + 100 μg/ml of chloramphenicol Gram staining, catalase production, gaseous requirements, biochemical test	*C*. *albicans* in primary lesions (8 isolates; 6.4%) Secondary lesions = 0	0
**Lysakowska et al. 2016** [76] **Poland**	33 Mean age 45.2 yrs; no systemic diseases; no antibiotics in the previous 6 months	37 (9 primary, 28 secondary) No periodontal pocket, no fracture involving pulp chamber; sample from the root with periapical radiolucency or largest canal	Pumice+water, caries removal, rubber dam isolation, disinfection (30% H2O2, 5.25% NaOCl, 5% Na_2_S_2_O_3_), sterility control swab sample, endodontic access; no chemical irrigant used.	Paper point for 1 min; placed in RTF; processed within 4 h	Columbia agar medium supplemented with 5% sheep’s blood and Enterococcosel Agar Biochemical test, chromogenic *Candida* differential agar	*C*. *albicans* in primary (1; 11.11%), Secondary lesions (1; 3.57%), Total (2; 5.41%)	0
**Bernal-Treviño et al. 2018** [79] **Mexico**	47	50 (36 primary, 14 secondary)	NS	(a) aspiration. (b) paper point	SDA; anaerobic enriched blood agar Macro- and micromorphology, germ-tube test, chromogenic differential agar, biochemical test	*Candida* spp. in primary lesions (15/36, 41.67%), Secondary lesions (3/14; 21.43%), Total (18/50; 36.00%)	2

BHI = brain heart infusion; CBA = Columbia blood agar; CHX = chlorhexidine; ETSA = enriched tryptic soy agar; F = females; FAA = fastidious anaerobe agar; H2O2 = hydrogen peroxide; HCB = yeast–cysteine blood agar; LDTM = liquid dental transport media; M = males; Min = minutes; Na2S2O3 = sodium thiosulfate; NaOCl = sodium hypochlorite; NS = not specified; NSAIDs = Nonsteroidal anti-inflammatory drugs; PBS = Phosphate-buffered saline; PRAS = pre-reduced anaerobically sterilized; R2A agar = Reasoner’s 2 agar; RCT = root canal treatment; RTF = reduced transport fluid; S = seconds; SDA = Sabourad dextrose agar; SDS-PAGE = sodiumdodecylsulphate polyacrylamide gel electrophoresis; spp. = species; TSBV = Tryptic Soy-serum-Bacitracin-Vancomycin; VMGA = Viability Medium Göteborg Agar; yrs = years. **RISK OF BIAS**: (0 low risk; 1–2 moderate risk; 3–5 high risk).

**Table 4:** Characteristics of the included studies using molecular methods for assessing fungal presence in teeth with primary endodontic infection.

Study Country	No. of subjects and characteristics	No. of samples and characteristics	Procedures before collection	Sample collection	Yeast identification method	Yeast spp. recovered (number of cases; prevalence)	Risk of bias
**Baumgartner et al. 2000** [44] **United States**	NS	24 teeth	Rubber dam isolation; disinfection (30% H2O2 for 1 min, 5% iodine for 1 min, 5% Na_2_S_2_O_3_); endodontic access (no water spray)	Paper points; placed in RTF and frozen at -70°C	Primer for *C*. *albicans*: **F**: CGA TTC AGG GGA GGT AGT GAC **R**: GGT TCG CCA TAA ATG GCT ACC AG Specificity of primer was tested against *P*. *nigrescens* (33563), *P*. *intermedia* (25611), *P*. *gingivalis* (33277), *P*. *endodontalis* (35406), *P*. *anaerobius* (27337)	*C*. *albicans* (5; 20.83%)	0
**Siqueira et al. 2002** [50] **Brazil**	NS age range 18–60 yrs	50 Single-rooted teeth	Pumice cleansing; rubber dam isolation; disinfection (3% H2O2, 2.5% NaOCl); endodontic access (no water spray); disinfection (2.5% NaOCl, 5% Na_2_S_2_O_3_); introduction of sterile saline into the canals if dried	(a) #15 K-type file (b) 2 paper points for 1 min; placed in TSB-DMSO and frozen at– 20°C	Fungal universal primer for ITS2 region: ITS3: GCA TCG ATG AAG AAC GCA GC **ITS4**: TCC TCC GCT TAT TGA TAT GC	Fungal species (1; 2%)	0
**Tsang et al. 2003** [53] **Hong Kong**	30 Southern Chinese	30 teeth	NS	NS	Primer for *C*. *albicans*: specific for *C*. *albicans* 70kDa-heat shock protein gene	*C*. *albicans* (1; 3.33%)	2
**Cogulu et al. 2008** [62] **Turkey**	66 Children; no systemic diseases; no antibiotics in the last 3 months	66 Permanent molars no recession, no periodontal pocket (only one root sampled: the one with periapical radiolucency or largest)	Pumice cleansing, rubber dam isolation, 3% H2O2 for 30 s, 2.5% NaOCl for 30 s, 5% Na_2_S_2_O_3_. Endodontic access (no water spray). No chemical irrigants. Introduction of sterile saline into the canals if dried	(a) #15 K-type file for 60 s (b) 2 paper points for 60 s. Placed in VMGA III transport medium, frozen at -20°C. Processed within 2 h	Primer for *C*. *albicans* toward *act1* gene for actin: **F**: GCC GGT GAC GAC GCT CCA AGA GCT G **R**: CCG TGT TCA ATT GGG TAT CTC AAG GTC Positive control strains: *C*. *albicans* ATCC 1023	*C*. *albicans* (2; 3.03%)	1
**Miranda et al. 2009** [64] **Brazil**	168 No antibiotic and antifungal treatment during the previous 6 months, no systemic diseases; 20-65yrs	184 Periapical radiolucency, no pulp exposure (only one root sampled from multi-rooted, widest)	Rubber dam isolation; endodontic access (no water spray), disinfection (30% H2O2 for 1 min, 5% iodine for 1 min, 5% Na_2_S_2_O_3_ for 1 min), sterility control sample; Introduction of sterile saline into the canals	3 paper points for 1 min, placed in modified Sabouraud broth with 100 mg L-1 chloramphenicol	PCR fingerprint technique. Primer: EI1: CTG GCT TGG TGT ATG T Positive control strains: *C*. *albicans* ATCC 18804, *C*. *parapsilosis* ATCC 22019, *C*. *krusei* ATCC 2159, *C*. *tropicalis* UFMG- A10, *C*. *glabrata* NCYC 388, *C*. *lusitaniae* CBS 6936, *C*. *dubliniensis* CBS 7987. Different genetic fingerprint profile was identified by sequencing the D1/D2 variable domains of the large subunit rDNA (primers: NL-1, NL-4)	Patient-level analysis: 16 teeth randomly excluded form analysis. *C*. *albicans* (34), *C*. *parapsilosis* (3), total *Candida* spp. (38; 22.6%)	0
**Rôças & Siqueira 2011** [67] **Brazil**	50 no antibiotics in the last 3 months	47 (3/50 excluded for positive sterility control samples) Single-rooted teeth, asymptomatic periapical lesion, no periodontal pocket, no gross carious lesion/fracture	Pumice cleansing, caries/restoration removal, rubber dam isolation, disinfection (3% H2O2, 2.5% NaOCl), endodontic access (sterile saline irrigation), disinfection (3% H2O2, 2.5% NaOCl, 5% Na_2_S_2_O_3_); sterility control samples (paper points). No chemical irrigants. Introduction of sterile saline into the canals if dried	3 paper points for 1 min; placed in TE buffer, frozen at -20°C	18S-rRNA genes amplification with fungal universal primer: B2f: ACT TTC GAT GGT AGG ATA G B4r: TGA TCR TCT TCG ATC CCC TA Positive control strains: *C*. *albicans* ATCC 10231	Fungal species 0	0
**Rôças & Siqueira 2011** [68] **Brazil**	27 no antibiotics in the last 3 months	24 (3/27 excluded for positive sterility control samples) Single-rooted teeth, asymptomatic periapical lesion, no periodontal pocket, no gross carious lesion/fracture	Pumice cleansing, caries/restoration removal, rubber dam isolation, disinfection (3% H2O2, 2.5% NaOCl), endodontic access (sterile saline irrigation), disinfection (3% H2O2, 2.5% NaOCl, 5% Na_2_S_2_O_3_); sterility control samples (paper points). No chemical irrigants. Introduction of sterile saline into the canals if dried	3 paper points for 1 min; placed in TE buffer, frozen at -20°C	18S-rRNA genes amplification with fungal universal primer: B2f: ACT TTC GAT GGT AGG ATA G B4r: TGA TCR TCT TCG ATC CCC TA Positive control strains: *C*. *albicans* ATCC 10231	Fungal species 0	0
**Brito et al. 2012** [70] **Brazil**	60 (40 HIV-, 20 HIV+) no antibiotics in the last 3 months, no periodontal pocket	60 (only the largest root associated with the lesion was sampled from multi-rooted)	Rubber dam isolation, disinfection (30% H2O2, 5% iodine, 5% Na_2_S_2_O_3_)	#10 K-type file; placed into alkaline lysis buffer	Checkerboard DNA-DNA Hybridization Positive control strains: *C*. *albicans* ATCC 10231, *C*. *tropicalis* ATCC 750	C. albicans (1 of 20 HIV+; 5% of HIV+; 1.67% overall)	1
**Paiva et al. 2012** [72] **Brazil**	30 no antibiotic therapy in the last 3 months, no periodontitis	27 (3 teeth out of 30 excluded for positive sterility control samples) Single-rooted teeth, asymptomatic periapical lesion, no periodontal pocket, no gross carious lesion/fracture	Pumice cleansing, caries/restoration removal, rubber dam isolation, disinfection (6% H2O2, 2% iodine, 6% H2O2, 2.5% NaOCl) endodontic access (sterile saline irrigation), disinfection (as above + 5% Na_2_S_2_O_3_); sterility control samples (paper points). No chemical irrigants. Introduction of sterile saline into the canals	5 paper points for 1 min; 2 placed in TE buffer and frozen at -20°C (other samples used for cultural analysis)	End-point PCR: 18S-rRNA genes amplification with primers: B2f: ACT TTC GAT GGT AGG ATA G B4r: TGA TCR TCT TCG ATC CCC TA Primer for *C*. *albicans*: **F**: GCC GGT GAC GAC GCT CCA AGA GCT G **R:** CCG TGT TCA ATT GGG TAT CTC AAG GTC Positive control strains: *C*. *albicans* ATCC 10321, ATCC 24433	Fungal species 0	0
**De Miranda & Colombo 2018** [78] **Brazil**	32 No antibiotic and/or anti-inflammatory therapy in the last 3 months, no diabetic patients	32 Apical periodontitis, mandibular first and second molars, no periodontal pocket	Rubber dam isolation, disinfection (30% H2O2, 5.25% NaOCl), caries/restoration removal, endodontic access, disinfection (as above)	3 paper points for 1 min; placed in TE buffer and frozen at -80°C	Checkerboard DNA-DNA hybridization Positive control strains: *C*. *albicans* ATCC 10231	*C*. *albicans* (15, 46.88%)	1
**De La Torre-Luna et al. 2019** [80] **Mexico**	120 60 T2DM, 60 non-diabetics, no other severe systemic diseases, no active periodontitis, no smokers, age range 22–69 yrs	120 Teeth with pulp necrosis, no sinus tract, single-rooted teeth, and mandibular molars (only distal canal sampled)	Caries/restoration removal, rubber dam isolation, disinfection (3% H2O2, 2.5% NaOCl) endodontic access, coronal flaring with 2.5% NaOCl irrigation, 5% Na_2_S_2_O_3_	(a) #15 hand file, placed in phosphate-buffered saline (b) 2 paper points, placed in phosphate-buffered saline, frozen at -80°C	Primer for *C*. *albicans*: **F**: GCC GGT GAC GAC GCT CCA AGA GCT G **R**: CCG TGT TCA ATT GGG TAT CTC AAG GTC Positive control strain: *C*. *albicans* ATCC 10231	*C*. *albicans* in T2DM patients (23, 38.3%); in non-diabetic (11, 18.3%)	1
**Zargar et al. 2020** [82] **Iran**	41 (20 irreversible pulpitis, 21 pulpal necrosis, no periodontal pocket > 4 mm); no severe systemic diseases, no antibiotic therapy in the last 30 days, M: f = 26:15, age range 18–60 yrs (mean age 33.8 ± 9.8 yrs)	41	Rubber dam isolation, disinfection (30% H2O2, 3% NaOCl), caries/restoration removal and endodontic access with sterile bur, disinfection (30% H2O2, 3% NaOCl, 5% Na_2_S_2_O_3_), sterility control sample	(a) #15 and #20 Hedstrom file; placed in thioglycolate (b) #20 and #25 paper point for 1 min; placed in thioglycolate	Primer for *C*. *albicans*: GCA TCG ATG AAG AAC GCA GCT CCT CCG CTT ATT GAT ATG C	*C*. *albicans* (11; 26.83%; 10 out of 11 cases had periapical involvement)	0

ATCC = American Type Culture Collection (Rockville, MD); °C = degree Celsius; F = forward; f = females; H_2_O_2_ = hydrogen peroxide; M = males; min = minutes; Na_2_S_2_O_3_ = sodium thiosulfate; NaOCl = sodium hypochlorite; NS = not specified; PCR = polymerase chain reaction; R = reverse; RCT = root canal treatment; RTF = reduced transport fluid; T2DM = type 2 diabetes mellitus; TE = Tris–ethylenediaminetetraacetic acid; TSB-DMSO = trypticase-soy broth with 5% dimethyl sulfoxide; VMGA = Viability Medium Göteborg Agar **RISK OF BIAS**: (0 low risk; 1–2 moderate risk; 3–5 high risk).

**Table 5:** Characteristics of the included studies using molecular methods for evaluating fungal presence in teeth with secondary endodontic infection.

Study Country	No. of subjects and characteristics	No. of samples and characteristics	Procedures before collection	Sample collection	Yeast identification method	Yeast spp. recovered (number of cases; prevalence)	Risk of bias
**Siqueira & Rôças 2004** [55] **Brazil**	22 Age range 29–80 yrs, mean age 46 yrs	22 RCT completed > 2 y earlier, no direct exposure to oral cavity, no periodontal pocket	Pumice cleansing, rubber dam isolation, 3% H2O2, 2.5% NaOCl. Endodontic access, 2.5% NaOCl, mechanical removal of root filling (no solvent). Introduction of sterile saline into the canals	(a) #15 K-type file (b) 3 paper points; placed in TE buffer and frozen at -20°C	Primer for *C*. *albicans* toward *act1* gene for actin: GCC GGT GAC GAC GCT CCA AGA GCT G CCG TGT TCA ATT GGG TAT CTC AAG GTC Positive control strains: *C*. *albicans* ATCC 10231, ATCC 44858	*C*. *albicans* (2; 9.09%)	0
**Rôças et al. 2008** [63] **Germany**	13 Age range 22–60 yrs, mean age 43.5 yrs	17 RCT completed at least 1 year earlier, no direct exposure to oral cavity	Rubber dam isolation, 3% H2O2, 3% NaOCl. Endodontic access, 3% NaOCl. Mechanical removal of root filling (no solvent). Introduction of sterile saline into the canals if dried	2 paper points; placed in TE buffer	Primer for *C*. *albicans*, toward *act1* gene for actin: **F**: GCC GGT GAC GAC GCT CCA AGA GCT G **R**: CCG TGT TCA ATT GGG TAT CTC AAG GTC Positive control strains: *C*. *albicans* ATCC 10231, ATCC 44858	*C*. *albicans* (1; 5.88%)	0
**Miranda et al. 2009** [64] **Brazil**	168 No atb and antifungal treatment during the previous 6 months, no systemic diseases; 20-65yrs	184 Periapical radiolucency, no pulp exposure (only one root sampled from multi-rooted, widest)	Rubber dam isolation; endodontic access (no water spray), disinfection (30% H2O2 for 1 min, 5% iodine for 1 min, 5% Na_2_S_2_O_3_ for 1 min), sterility control sample; Introduction of sterile saline into the canals	3 paper points for 1 min, placed in modified Sabouraud broth with 100 mg L-1 chloramphenicol	PCR fingerprint technique. Primer: EI1: CTG GCT TGG TGT ATG T Positive control strains: *C*. *albicans* ATCC 18804, *C*. *parapsilosis* ATCC 22019, *C*. *krusei* ATCC 2159, *C*. *tropicalis* UFMG- A10, *C*. *glabrata* NCYC 388, *C*. *lusitaniae* CBS 6936, *C*. *dubliniensis* CBS 7987. Different genetic fingerprint profile was identified by sequencing the D1/D2 variable domains of the large subunit rDNA (primers: NL-1, NL-4)	Patient-level analysis: 16 teeth randomly excluded form analysis. *C*. *albicans* (34) *C*. *parapsilosis* (3), Total *Candida* spp. (37, 22.6%)	0
**Anderson et al. 2012** [69] **Germany**	21 no systemic diseases, no atb in the last 30 days	20 (1 tooth out of 21 excluded for contamination of quality control sample) RCT completed at least 2 yrs earlier, no direct exposure to oral cavity, asymptomatic	Rubber dam isolation, disinfection (30% H2O2, 2,5% NaOCl), endodontic access, disinfection (30% H2O2, 2,5% NaOCl, 5% Na_2_S_2_O_3_), sterility control samples (foam pellets), mechanical removal of root filling (no solvent). Introduction of sterile saline into the canals	3 paper points for 1 min; placed in RTF, frozen at -20°C	18S-rRNA genes amplification with primers: **ITS1**: CTT GGT CAT TTA GAG GAA GTA A **ITS4**: TCC TCC GCT TAT TGA TAT GC	Fungal species (1; 5%)	0
**Dumani et al. 2012** [71] **Turkey**	170 (100 primary, 70 secondary) M: f = 63:107	231 (117 primary, 114 secondary)	Pumice cleansing, rubber dam isolation, disinfection (35% H2O2, 5% NaOCl), endodontic access, disinfection (5% NaOCl, Na_2_S_2_O_3_), sterility control samples (cotton pellets), eventually mechanical removal of root filling (no solvent), introduction of sterile saline into the canals	3 paper points for 1 min; placed in TE buffer, frozen at -20°C	Primer for *C*. *albicans* toward *act1* gene for actin: **F**: GCC GGT GAC GAC GCT CCA AGA GCT G **R**: CCG TGT TCA ATT GGG TAT CTC AAG GTC Positive control strains: *C*. *albicans* ATCC 90028	Primary: *C*. *albicans* (23; 19.66%) Secondary: *C*. *albicans* (13; 11.40%)	0
**Poptani et al. 2012** [73] **India**	NS no antibiotic therapy in the last 1 month	20 Symptomatic; RTC completed at least 1 yr. earlier, 1 root per tooth was sampled (the one associated to lesion and/or widest)	Pumice cleansing, rubber dam isolation, disinfection (3% H2O2, 2.5% NaOCl), endodontic access (with saline irrigation), disinfection (2.5% NaOCl), mechanical removal of root filling (no solvent), introduction of sterile saline into the canals	2–3 paper points for 1 min; placed in TE buffer and frozen at -20°C	Primer for *C*. *albicans*: f: GCC GGT GAC GAC GCT CCA AGA GCT G r: CCG TCA GGG GAC GTT CAG Positive control strains: *C*. *albicans* ATCC 10231	*C*. *albicans* (7; 35%)	0
**Karygianni et al. 2015** [75] **Germany**	5 No periodontitis, no severe systemic diseases, no antibiotic therapy in the last 30 days	5 First lower molars, asymptomatic, RCT completed at least 2 yrs earlier, no direct exposure to oral cavity	Disinfection (30% H2O2, 2.5% NaOCl), rubber dam isolation, endodontic access, disinfection (30% H2O2, 2.5% NaOCl, 5% Na_2_S_2_O_3_), sterility control sample, mechanical root-filling removal (no solvents), introduction of sterile saline into the canal	a) Obturation material samples; placed in RTF (b) 3 paper points for 1 min; placed in RTF and frozen at -80°C	18S-rRNA genes amplification with primers: **ITS1**: CTT GGT CAT TTA GAG GAA GTA A **ITS4**: TCC TCC GCT TAT TGA TAT GC	Fungal species 0	1
**Zargar et al. 2019** [81] **Iran**	30 No severe systemic diseases, no antibiotic therapy in the last 30 days, age range 23–70 yrs	30	Disinfection (30% H2O2, 3% NaOCl), caries/restoration removal and endodontic access with sterile bur, rubber dam isolation, disinfection (30% H2O2, 3% NaOCl, 5% Na_2_S_2_O_3_), sterility control sample; mechanical root-filling removal (no solvents). introduction of sterile saline into the canal	(a) #20 and #25 Haedstrom file; placed in thioglycolate (b) #20 and #25 paper point for 1 min; placed in thioglycolate	Primer: **ITS3**: GCA TCG ATG AAG AAC GCA GC	*C*. *albicans* (10; 33.33%)	0

Atb = antibiotics; ATCC = American Type Culture Collection (Rockville, MD); °C = degree Celsius; F = forward; f = females; H_2_O_2_ = hydrogen peroxide; M = males; min = minutes; Na_2_S_2_O_3_ = sodium thiosulfate; NaOCl = sodium hypochlorite; NS = not specified; PCR = polymerase chain reaction; R = reverse; RCT = root canal treatment; RTF = reduced transport fluid; spp. = species; T2DM = type 2 diabetes mellitus; TE = Tris–ethylenediaminetetraacetic acid; TSB-DMSO = trypticase-soy broth with 5% dimethyl sulfoxide; VMGA = Viability Medium Göteborg Agar; yrs = years. **RISK OF BIAS**: (0 low risk; 1–2 moderate risk; 3–5 high risk)

**Table 6:** Characteristics of the included studies assessing prevalence of yeasts in both primary and secondary endodontic infections, using molecular methods.

Study Country	No. of subjects and characteristics	No. of samples and characteristics	Procedures before collection	Sample collection	Yeast identification method	Yeast spp. recovered (number of cases; prevalence)	Risk of bias
**Dumani et al. 2012** [71] **Turkey**	170 (100 primary, 70 secondary) M: f = 63:107	231 (117 primary, 114 secondary)	Pumice cleansing, rubber dam isolation, disinfection (35% H2O2, 5% NaOCl), endodontic access, disinfection (5% NaOCl, Na_2_S_2_O_3_), sterility control samples (cotton pellets), eventually mechanical removal of root filling (no solvent), introduction of sterile saline into the canals	3 paper points for 1 min; placed in TE buffer, frozen at -20°C	Primer for *C*. *albicans* toward *act1* gene for actin: **F**: GCC GGT GAC GAC GCT CCA AGA GCT G **R**: CCG TGT TCA ATT GGG TAT CTC AAG GTC Positive control strains: *C*. *albicans* ATCC 90028	Primary: *C*. *albicans* (23; 19.66%) Secondary: *C*. *albicans* (13; 11.40%)	

ATCC = American Type Culture Collection (Rockville, MD); °C = degree Celsius; F = forward; f = females; H_2_O_2_ = hydrogen peroxide; M = males; min = minutes; Na_2_S_2_O_3_ = sodium thiosulfate; NaOCl = sodium hypochlorite; R = reverse; RCT = root canal treatment; TE = Tris–ethylenediaminetetraacetic acid. **RISK OF BIAS**: (0 low risk; 1–2 moderate risk; 3–5 high risk).

**Fig 3:**
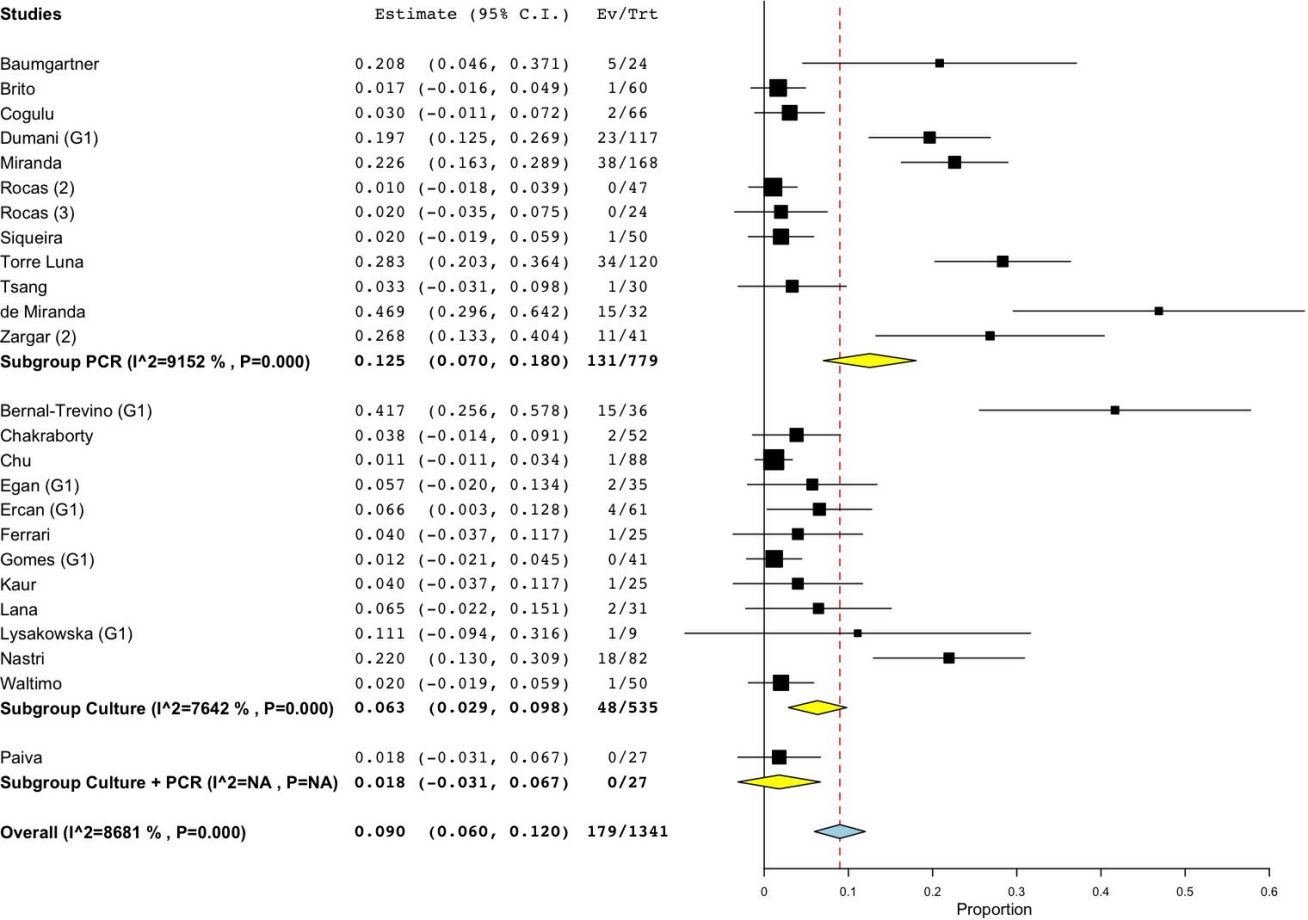
Forest plot representing fungal species prevalence in primary endodontic infections, distinguishing the methods of detection.

As for studies using cultural methods, a total of 535 specimens was analyzed: the prevalence of fungi ranged from 0 [54,72] to 41.67% [79] and the WMP was 6.3% (CI 95%: 2.9–9.8) (Fig 3). On the other hand, when molecular biological methods were used, the prevalence ranged from 0.00 to 46.88% [78], yielding a WMP of 12.5% (CI 95%: 7–18) in a total of 779 samples (Fig 3). When both techniques were combined, then the WMP was 1.8% [72] (Fig 3).

The studies that reported higher yeast prevalence [78,79] were based only on a small sample size (36 and 32 samples, respectively), while three of the studies with the highest number of clinical samples were based on PCR techniques and reported a prevalence of 19.66% [71], 22.62% [64] and 28.33% [80].

**Fig 4:**
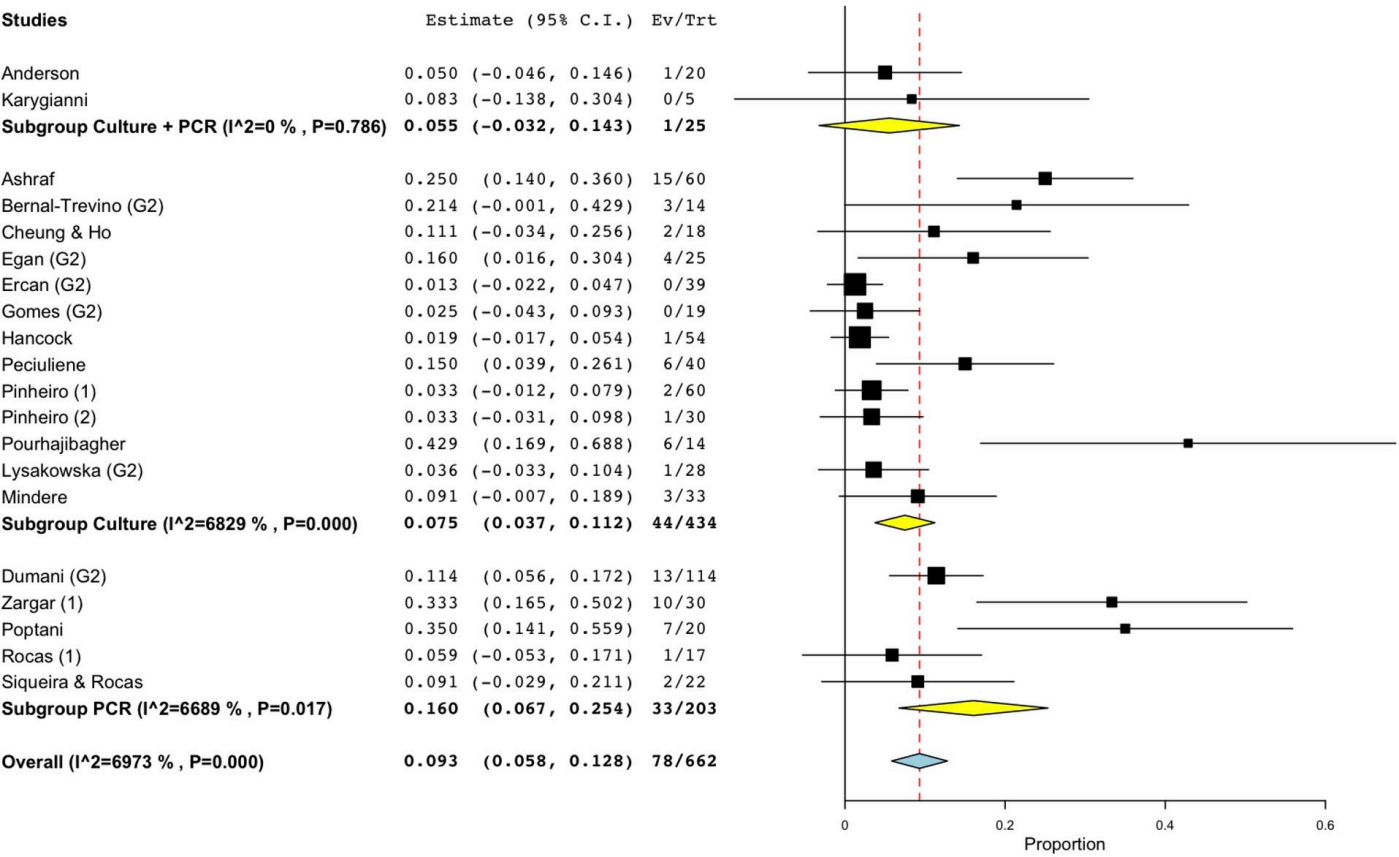
Forest plot representing fungal species prevalence in secondary endodontic infections, distinguishing the methods of detection.

As for persistent infections, the prevalence of fungi was reported to range between 0.00 and 42.86% [77], and the calculated WMP was 9.3% (CI 95%: 5.8–12.8) from a total of 662 samples (Fig 2). Cumulative culture data indicate that 434 specimens yielded 44 positive samples. The resultant WMP of yeasts was 7.5% (CI 95%: 3.7–11.2). In one case, a higher prevalence rate (42.86%) was found in a cohort of only six positive specimens, that may have led to high level of skewing of the results [77]. When PCR was used, the calculated WMP was 16.0% (CI 95%: 6.7–25.4) (Fig 4). In two studies [69,75], both techniques were used, resulting in a WMP of 5.5% (CI 95%: -3.2–14.3) (Fig 4).

Considering all endodontic infections evaluated, the mean prevalence of fungi was found to be 9.11% (CI 95%: 6.8–11.4) in a total of 2003 specimens. The foregoing clearly implies that fungi contribute to up to one in ten to one in eleven root canal infections and re-infections. Interestingly, no differences in WMP were found among the detecting methods for each lesion type (either primary or secondary).

**Fig 5:**
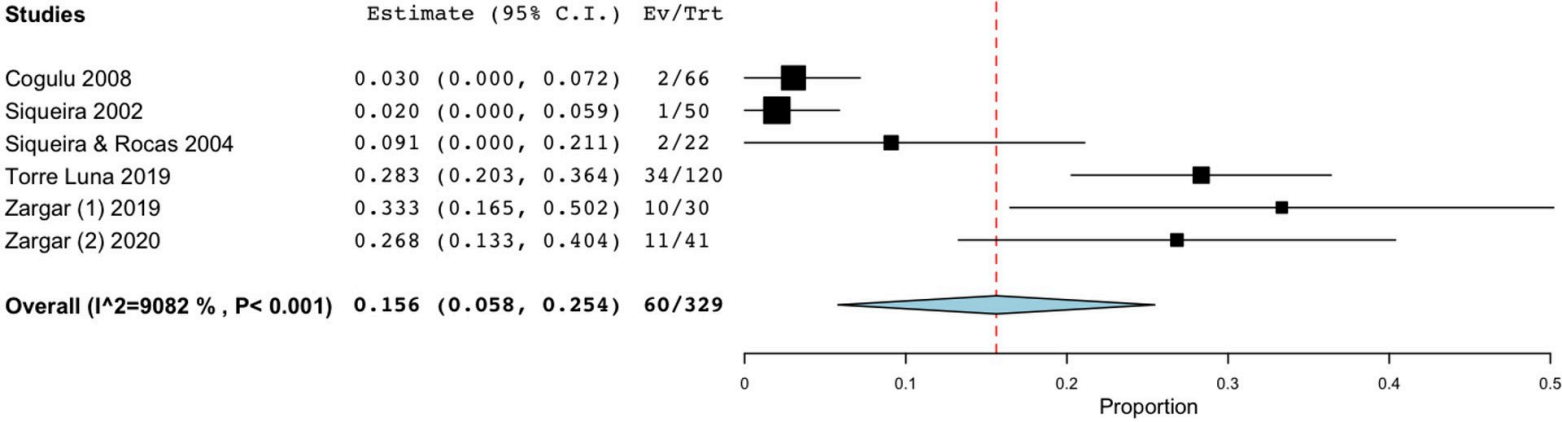
Forest plot representing fungal species prevalence in studies using both paper point and hand files for sample collection from teeth with primary and secondary endodontic infections.

**Fig 6:**
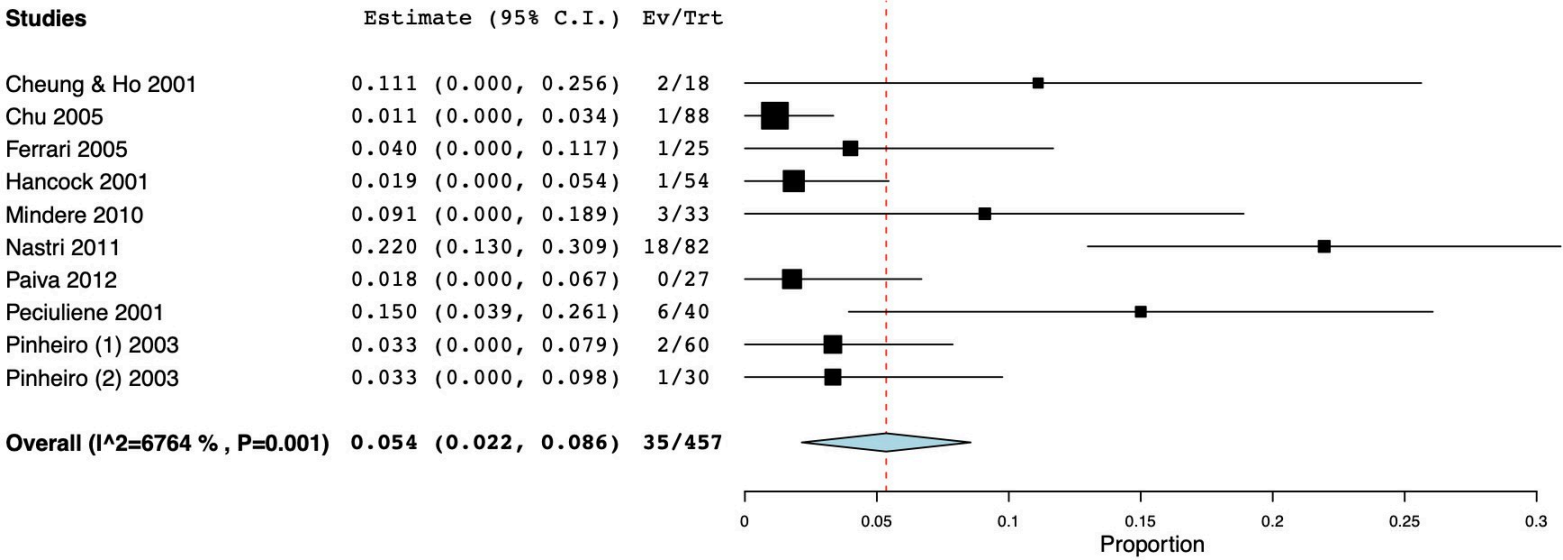
Forest plot representing fungal species prevalence in cultural studies evaluating teeth with primary and secondary endodontic infections with periapical lesions.

**Fig 7:**
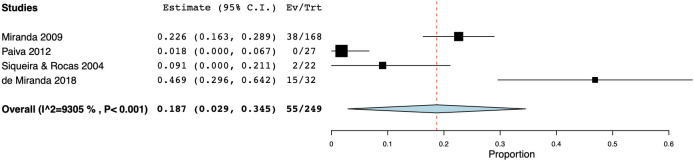
Forest plot representing fungal species prevalence in molecular studies assessing teeth with primary and secondary endodontic infections with periapical lesions.

**Fig 8:**
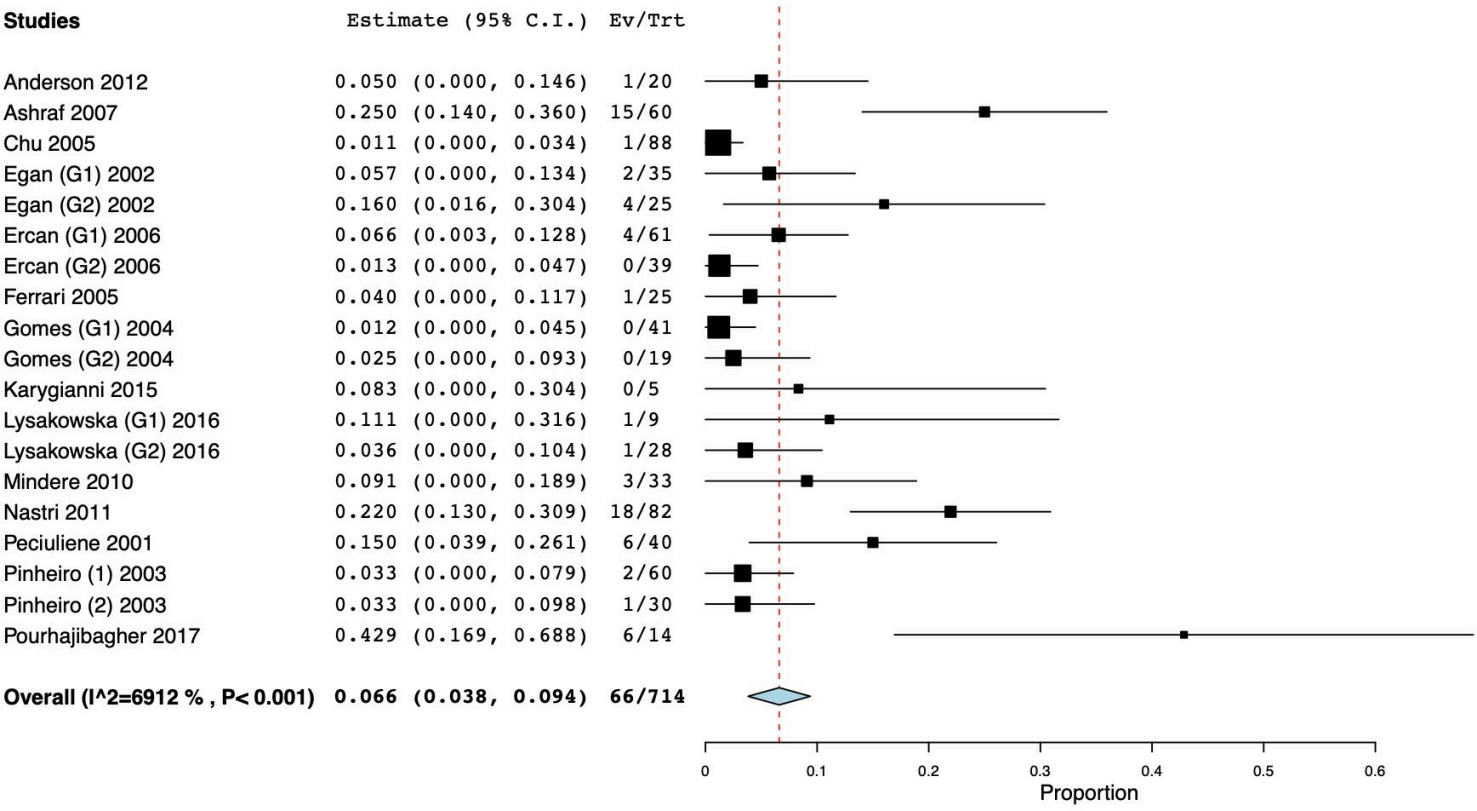
Forest plot representing fungal species prevalence in teeth with primary and secondary endodontic infections assessed via cultural methods in systemically healthy subjects.

**Fig 9:**
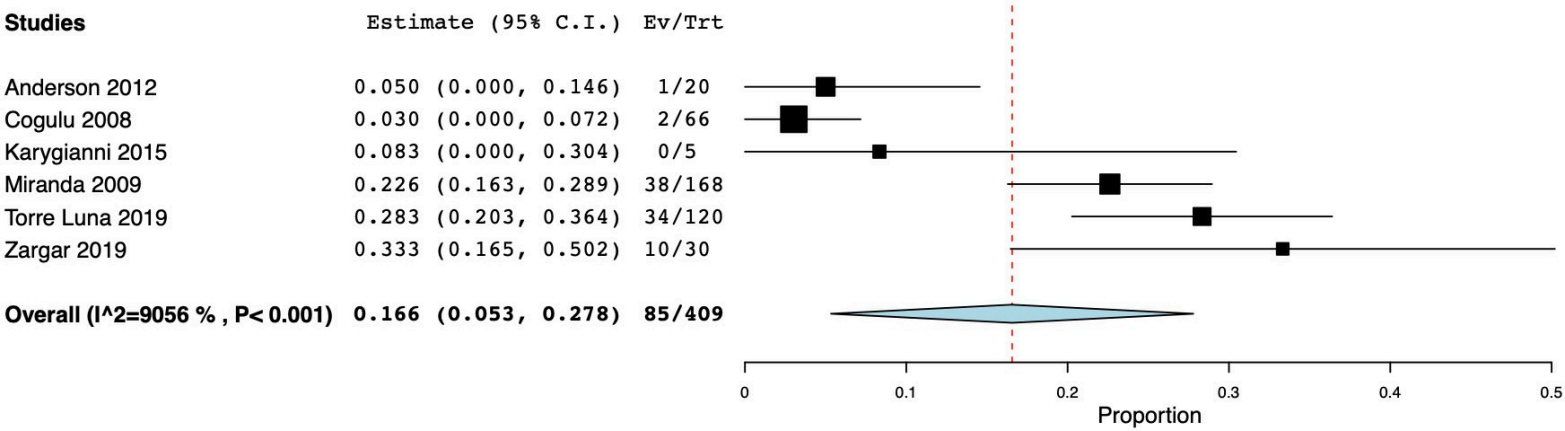
Forest plot representing fungal species prevalence in teeth with primary and secondary endodontic infections assessed via molecular techniques in systemically healthy subjects.

A total of seven workers [45,50,55,62,80–82] used both paper points and hand files for sample collection. Only one of them [45] used cultural methods, thus the meta-analysis could not be performed, while six of them [50,55,62,80–82] used PCR, and the resulting WMP was 15.6% (CI 95%: 5.8–25.4) (Fig 5). Thirteen studies only included teeth presenting periapical lesion, with a WMP of 5.4% (CI 95%: 2.2–8.6) for cultural studies [45,47,48,51,52,57,58,65,66,72] (Fig 6) and 18.7% (CI 95%: 2.9–34.5) for molecular studies [55,64,72,78] (Fig 7). Studies including only systemically healthy subjects were also separately analyzed, and the calculated WMP for the latter group was 6.6% (CI 95%: 3.8% - 9.4%) for 15 cultural studies, and 16.6% (CI 95%: 5.3–27.8) for six molecular studies (Figs 8 and 9).

The majority of the studies evaluated were deemed to be at low risk of bias, while 10 studies were considered as moderate risk. In most cases, the risk was associated to an incomplete description of the target population, and the lack of randomization of the samples, which could undermine the external validity of the results. For example, only mandibular molars [75,78], permanent teeth from pediatric patients [62], symptomatic [73] or asymptomatic teeth [48,58,66–69,72,75], or a limited age range were included [74], and in several studies that may have led to inherent bias. On the other hand, there were inadequate number of studies in each of the foregoing subgroups to conduct metanalyses.

In several other cases, the augmented risk of bias was increased due to poor or total absence of the description of the tooth and the sample site preparative procedures prior to the microbiological sampling [47,53,54,74,79]. On the contrary, some studies provided very precise descriptors of the protocols for sample selection (e.g. collection from the largest root canal associated with the periapical lesion) including the quality of disinfection of the sampling site, and single or multiple control specimens [45,46,48,57–59,64,67–69,71,72,75,76,81,82] all of which affect the microbiological outcomes. It must also be noted that all the included studies used rubber dam isolation procedures of the tooth before sampling procedures.

## Discussion

Our meta-analysis of the literature comparing the fungal biome of primary and secondary endodontic infections, comprised selectively curated 39 studies, performed over a period of approximately two decades. These indicate the presence of fungal infection in approximately one in ten endodontic infections, with similar prevalence rates in both the primary (9.0%) and secondary infections (9.3%). A great heterogeneity was observed in the analyzed data, which represents a limitation of the present study. There are a number of reasons for the heterogeneity of the data that were noted, and they are essentially dichotomous in nature and falls into two broad categories, the health and demographic characteristics of the studied subjects, and the microbiological sampling and evaluation technology.

The vast majority of the cases included in the review were from systemically healthy and immunocompetent adults, not undergoing antibiotic or antifungal therapy over 1–6 months prior to sampling. However, a minority of studies were in children [62], and some were in human immunodeficiency virus (HIV) infected [70], and diabetic subjects [80]. These accounted for a limited number of cases (146 samples, respectively from 66 children, 20 HIV positive and 60 diabetic patients). Interestingly, the diabetic patients presented with a high prevalence of *C*. *albicans* (38.3%) in the infected root canals [80].

The microbiological sampling and evaluation technology appear to have played a critical role in the disparate outcomes reported here irrespective of whether they were based on traditional microbial culture technology, or molecular biological studies. Culture or molecular technology outcomes are essentially a direct reflection of the sample quality [83], which can be significantly impacted by the poor isolation of the sampling site, inadequate asepsis and disinfection of the entry portal (of the sampling material), poor access cavity design, missed or obtuse canals, inadequate instrumentation and debridement to remove superficial debris and/or saliva contamination, and prior contamination of the root canal system due to seepage of the contaminant oral flora through poorly sealed temporary or permanent restorations (in the case of secondary infections).

As regards the sample collection protocols, multiple differences were found among studies. Paper point absorption method was the most commonly used, and proved to be the more sensitive according to one group [45], although endodontic instruments such as K-type or Headstrom files were used for sample collection by a large number of workers [45,50,55,61,62,70,80–82]. There were inherent deficiencies in some studies as chemicals, that may affect fungal viability, were used during the mechanical instrumentation by the researchers, prior to sample collection [80]. As for persistent chronic infections, no chemical solvents were used by any of the investigators, to remove the root filling except in one study by Egan et al. [49].

Varying protocols for field decontamination were used prior to the endodontic access by different workers, including the use of iodine, hydrogen peroxide, chlorhexidine gluconate, sodium hypochlorite, or isopropyl alcohol of varying dilutions [45,49,50,55,57,61,63,64,67–69,71–73,75,78,81,82]. It must be noted that liquid disinfectants may flow into the pulp chamber and interfere with the sampling if applied after endodontic access or when the endodontic space communicates with the oral cavity. Sodium thiosulfate was also used in some studies to inactivate iodine, so as not to impact the microbiological sampling quality. Some of the protocols also employed sterility checks using a control samples to ascertain the integrity of the sampling technique [45,46,48,57–59,64,67–69,71,72,75,76,81,82].

Another noteworthy point is the microbiological culture methods used by different investigators which may lead to discrepant results. Due to the quantitatively low numbers of fungi populating the infected root canal systems their growth may take upto 72 hr to be discernible as colony forming units (CFU) unless appropriate culture media are used and observed for at least three days. If the laboratory workers do not use fungal culture media (such as Sabourauds agar or chromogenic agar) and/or evaluates the culture results only upto 24 hr, then there is a high likelihood that the yeast prevalence will be under reported. However, this deficiency could be overcome through molecular mycological analyses using fungal specific universal primers, when the total culturable as well as unculturable fungal flora could be detected with confidence, irrespective of their population size within the endodontic ecosystem.

Another limitation of the study is that we were not able to perform a patient-level analysis of the prevalence of fungi in endodontic infections. Although the majority of the included studies collected and analyzed only one sample per patient, some of them included more samples from the same subjects [49,57,60,64,71,76,79], while in some other papers this was not specified [44,46,50–52,56,59,61,63,65,73].

Microorganisms may still persist within the root canal system even after adequate debridement and disinfection and related clinical procedures. This is due to the complex anatomy and the architecture of the root canal system that may include the presence of accessory canals, anatomical lacunae, and delta spaces. A number of workers have compared the prevalence of fungi in primary endodontic infections before and after endodontic therapy, and some have noted the increased prevalence of yeasts and other fungal species, post treatment, even though, in a few cases, they were undetectable prior to treatment [46,51,58]. The same findings had been already reported in previous studies [84,85]. This implies that fungi have the potential to often survive a harsh, nutritionally depleted and chemically infused ecosystem consequential to endodontic treatment procedures [86] and may gain access into the root canal system during or after the endodontic treatment.

Various studies have analyzed the inter kingdom co-habitation of yeasts and bacteria in the infected endodontic ecosystems. Although *C*. *albicans* is typically isolated from persistent endodontic infections in mixed cultures of cohabitant oral bacteria, at least four groups have reported identification of pure cultures of *Candida* from root canal infections [36,46,87,88]. Thus, Hancock et al. in a very early report, over two decades ago, described the mono-infection of root canals with *C*. *albicans* [45]. Also, in one of the largest studies to date, Waltimo and coworkers reported isolation of fungi in pure cultures from six of 967 endodontic samples, and yeast/bacterial mixed cultures in another 41 cases [86].

Another major reason for the heterogeneity of microbiological results we noted, could be the shift from cultural studies to molecular biological studies for microbiome evaluation, over the last few decades. Together with the increased sophistication of the molecular technologies there has been a parallel increase in the knowledge of the oral microbiome, leading to the elucidation of a hitherto unknown universe of unculturable flora. Accordingly, the cumulative data from our review clearly indicate that PCR analytical techniques yield higher fungal prevalence values as compared with the culture methods. This is clearly seen in WMP for fungi in primary and secondary infections which were 6.3% and 7.5% for pure culture-based studies, that increased to 12.5% and 16.0% in PCR studies, respectively. Adding to this confusion, some workers used both cultural and molecular techniques for mycological analyses, and additional positive cases were detected through PCR analyses [69,72,75]. This was also the case in a recent study by Al-Sakati et al. [88], who compared culture-dependent and culture-independent methods to detect bacteria and fungi in reinfected root-filled teeth, and found a higher detection rate of fungi using the latter techniques.

Apart from the low population density of fungi in infected root canal systems, there may be other reasons for heterogeneous results even within molecular mycological analyses. These include the use of dissimilar primers by different investigators, with varying degrees of specificity. Al-Sakati et al. [88] noted, for instance, an increased rate of fungal detection by improving the specificity of the primers used. One disadvantage of the use of multiple specific primers instead of a single universal primer is the increased costs and time associated with the former approach. Recently, matrix-assisted laser desorption ionization time-of-flight mass spectrometry (MALDI-ToF MS) has been proposed for fungal identification [89]. The absence of a comprehensive and well-curated spectral database of fungi, is the major limitation of this technique, although they are becoming increasingly robust and complete as time progresses with input from ongoing studies. MALDI-ToF MS analyses being inexpensive, easy, fast and accurate, and the fact that it can be applied to detect a comprehensive array of microorganism implies that the method would be very useful for future workers investigating the endodontic mycobiome. We reviewed, two studies which compared MALDI-ToF MS analysis and conventional cultural tests, but these workers failed to detect any fungal isolates from the endodontium [69,75].

Another exciting development in the quantitative and qualitative microbiological analyses is the advent of the Next Generation Sequencing (NGS) platforms. These, based on various platforms such as Illumina and Ion-torrent, as well as novel inexpensive, miniaturized platforms such as Minion technology, hold much promise in the demystification of the fungal flora of infected root canal systems.

## Conclusions

The role of fungi in the pathogenesis of endodontic infections is unclear, as yet. The significant heterogeneity of results in the reviewed studies reflect the variety of the techniques used for sample collection, culture and identification. On the other hand, novel molecular techniques such as MALDI-TOF and NGS analyses should elucidate the true mycobiome as the new technology could redefine both the culturable and unculturable mycobiota in endodontic infections. Finally, as almost one in ten endodontic infections are associated with fungal infestation, clinicians must pay heed the need to eradicate both bacteria and fungi from infected root canal systems during treatment procedures.

## Supporting information

S1 Checklist PRISMA 2009 checklist. (DOC)
